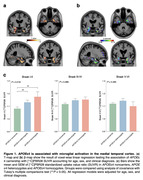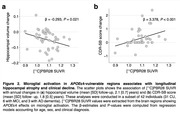# APOEε4 drives microglial activation in the medial temporal cortex in individuals across the AD spectrum

**DOI:** 10.1002/alz.088479

**Published:** 2025-01-09

**Authors:** João Pedro Ferrari‐Souza, Firoza Z Lussier, Douglas Teixeira Leffa, Joseph Therriault, Cécile Tissot, Bruna Bellaver, Pamela C.L. Ferreira, Guilherme Povala, Andrea L. Benedet, Stijn Servaes, Jenna Stevenson, Nesrine Rahmouni, Arthur C. Macedo, Jean‐Paul Soucy, Serge Gauthier, Diogo O. Souza, Eduardo R. Zimmer, Pedro Rosa‐Neto, Tharick Ali Pascoal

**Affiliations:** ^1^ University of Pittsburgh, Pittsburgh, PA USA; ^2^ Universidade Federal do Rio Grande do Sul, Porto Alegre, Rio Grande do Sul Brazil; ^3^ McGill University, Montreal, QC Canada; ^4^ Lawrence Berkeley National Laboratory, Berkeley, CA USA; ^5^ University of Gothenburg, Gothenburg Sweden; ^6^ Montreal Neurological Institute, McGill University, Montréal, QC Canada; ^7^ Universidade Federal do Rio Grande do Sul, Porto Alegre Brazil

## Abstract

**Background:**

Microglial activation is an early phenomenon in Alzheimer’s disease (AD) that may occur prior to and independently of amyloid‐β (Aβ) aggregation. Compelling experimental evidence suggests that the apolipoprotein E ε4 (*APOE*ε4) allele may be a culprit of early microglial activation in AD. However, it is unclear whether the *APOE*ε4 genotype is associated with microglial reactivity in the living human brain. In individuals across the aging and AD spectrum, we tested the hypothesis that *APOE*ε4 associates with microglial activation.

**Method:**

We studied 118 individuals (79 cognitively unimpaired [CU], 23 with mild cognitive impairment [MCI], and 16 with AD dementia) from the Translational Biomarkers in Aging and Dementia (TRIAD) cohort. Individuals had available [^18^F]AZD4694 Aβ PET, [^18^F]MK6240 tau PET, [^11^C]PBR28 microglial activation PET, and magnetic resonance imaging (MRI), as well as *APOE* genotyping. To increase the reliability of our results, we only included high‐affinity binders for the [^11^C]PBR28 radiotracer. In a subgroup of 42 individuals with longitudinal clinical and MRI data, we further assessed longitudinal hippocampal atrophy and clinical deterioration.

**Result:**

Voxel‐wise analysis revealed that *APOE*ε4 carriership was associated with increased [^11^C]PBR28 uptake mainly in the medial temporal cortex (Figure 1A and B), and this effect of *APOE*ε4 was independent of Aβ and tau accumulation. Region‐wise analyses demonstrated that *APOE*ε4 carriers presented increased [^11^C]PBR28 SUVR relative to noncarriers only in Braak I‐II regions (Figure 1C), which further supports that *APOE*ε4‐related microglial activation occurs specifically in medial temporal structures. Lastly, we found that [^11^C]PBR28 uptake in brain regions vulnerable to *APOE*ε4 effects is associated with subsequent hippocampal atrophy and clinical decline over 2 years (Figure 2).

**Conclusion:**

These results support a model in which APOEε4 plays a role in early AD progression by contributing to microglial activation in medial temporal regions. Our findings provide a rationale for the development of novel AD therapies targeting the interplay between ApoE and neuroinflammation.